# The significance of the crosstalk between ubiquitination or deubiquitination and ncRNAs in non-small cell lung cancer

**DOI:** 10.3389/fonc.2022.969032

**Published:** 2023-01-16

**Authors:** Yiyang Sun, Ping He, Li Li, Xue Ding

**Affiliations:** ^1^ Department of Gerontology and Geriatrics, Shengjing Hospital of China Medical University, Shenyang, China; ^2^ General Medicine Department, Dalian Friendship Hospital, Dalian, China

**Keywords:** miRNA, lncRNA, circRNA, ubiquitination, deubiquitination, NSCLC

## Abstract

Lung cancer (LC) remains the leading cause of cancer-related deaths worldwide, with extremely high morbidity and mortality rates. Non-small cell lung cancer (NSCLC) is the most critical type of LC. It seriously threatens the life and health of patients because of its early metastasis, late clinical symptoms, limited early screening methods, and poor treatment outcomes. Non-coding RNAs (ncRNAs), including microRNAs (miRNAs), long non-coding RNAs (lncRNAs), and circular RNAs (circRNAs), participate in cell proliferation, metastasis, and chemoresistance. Several previous studies have proven that ncRNAs are vital regulators of tumorigenesis. Ubiquitination plays the most crucial role in protein post-translational modification (PTM). Deubiquitination and ubiquitination form a homeostasis. In summary, ubiquitination and deubiquitination play essential roles in mediating the degradation or overexpression of a range of crucial proteins in various cancers. A growing number of researchers have found that interactions between ncRNAs and ubiquitination (or deubiquitination) play a crucial role in NSCLC. This review presents several typical examples of the important effects of ncRNAs and ubiquitination (or deubiquitination) in NSCLC, aiming to provide more creative ideas for exploring the diagnosis and treatment of NSCLC.

## 1 Introduction

Many well-known international studies have confirmed that cancer is the primary cause of premature death. Lung cancer still ranks first in terms of fatality rate (18.0%) and is the second most diagnosed cancer (11.4%) (1).Based on histopathological characteristics, lung cancer can be divided into small-cell lung cancer (SCLC) and non-small cell lung cancer (NSCLC). NSCLC accounts for up to 85% of lung cancer (2). The latest advances in diagnostic tools and systematic treatment methods have improved the therapeutic effect of NSCLC, with a 5-year survival rate of 33% for regional-stage disease and 60% for localized-stage disease. However, owing to the characteristics of early metastasis and late clinical symptoms, the 5-year survival rate of patients with distant metastasis is only 6% (3). Therefore, early detection, diagnosis, and treatment are essential for prolonging the survival time of NSCLC patients.

The Human Genome Project found that genes encoding proteins account for only 2% of the human genome, and most of the remaining human transcriptomes are ncRNAs (4). The vast number of ncRNAs was once considered non-functional “garbage” until the ENCODE project showed that the non-protein-coding parts of genes could be copied into thousands of RNA molecules, which regulate fundamental biological processes and play vital roles in the entire human disease spectrum. However, the boom in ncRNA research has only begun (5). There are many types of ncRNAs, and current research mainly focuses on microRNAs (miRNAs), long non-coding RNAs (lncRNAs), and circular RNAs (circRNAs).

MiRNAs are small RNAs of approximately 22 nucleotides that regulate the expression of specific genes by pairing them completely or incompletely with the 3’-UTR region of the mRNA of the target genes to degrade mRNAs or inhibit their post-transcriptional translation (6). MiRNAs are typically processed in the nucleus by RNA polymerase II (polII) and Drosha as precursor miRNAs (pri-miRNAs), which are then exported to the cytoplasm and cleaved into double-stranded RNA by the RNase III enzyme, Dier. One of the strands was then selected and incorporated into a miRNA-induced silencing complex (miRISC). Complementary miRNA and mRNA usually lead to the degradation of the target mRNA. However, when not entirely complementary, miRNAs usually prevent the expression of target genes at the protein level without influencing mRNA stability (7). MiRNAs are often heavily dysregulated and function as oncogenes or tumor suppressors in cancer cells. MiRNAs can act as diagnostic and prognostic markers, which bring vast attention to cancer diagnosis and treatment (8, 9).

LncRNAs are non-coding RNAs that are longer than 200 nucleotides and have 5’-modified caps and 3’-polyadenylated tails. LncRNAs are closely related to their cellular localization and play essential roles in almost all phases of gene regulation (10). LncRNAs affect chromatin and transcription levels in the nucleus, leading to post-transcriptional regulation in the cytoplasm. In addition, they regulate fine-tuning of the translation process and RNA molecules directly or indirectly by affecting the expression of upstream or downstream genes (11). In terms of mechanisms of action, decoy lncRNAs can bind proteins or RNAs, resulting in the negative regulation of protein expression. In addition, guide lncRNAs direct protein localization by binding to proteins and signal lncRNAs to interact with transcription factors or chromatin-modifying enzymes, resulting in the regulation of transcription and signaling pathways. Lastly, scaffold lncRNAs act as organizing structures where molecules can bind and interact with each other more efficiently (12). Aberrant expression of numerous lncRNAs participates in several types of carcinogenesis.

CircRNAs are a class of endogenous RNAs that regulate gene expression, and the 3’ and 5’ ends of circRNAs are covalently bound by trans-splicing to form a closed circular structure. Unlike linear RNA structures, circRNAs do not have 5’-3’ polarity or polyadenylated tails, which makes them more stable (13). As a result, circRNAs can resist the decomposition of RNase enzymes and have higher sequence conservation, abundance, and tissue specificity (14). CircRNAs mainly work through four molecular mechanisms. First, circRNAs can act as competing endogenous RNAs (ceRNAs) of miRNAs. Second, circRNAs interact with RNA-binding proteins (RBPs) to regulate the mRNA of many genes. Third, a balance exists between linear RNAs and circRNAs through competitive complementary pairing. Fourth, although circRNAs are non-coding RNAs, some circRNAs can perform regulatory functions through translation (15). CircRNAs affect all aspects of the occurrence and development of different cancers.

**Figure 1:**
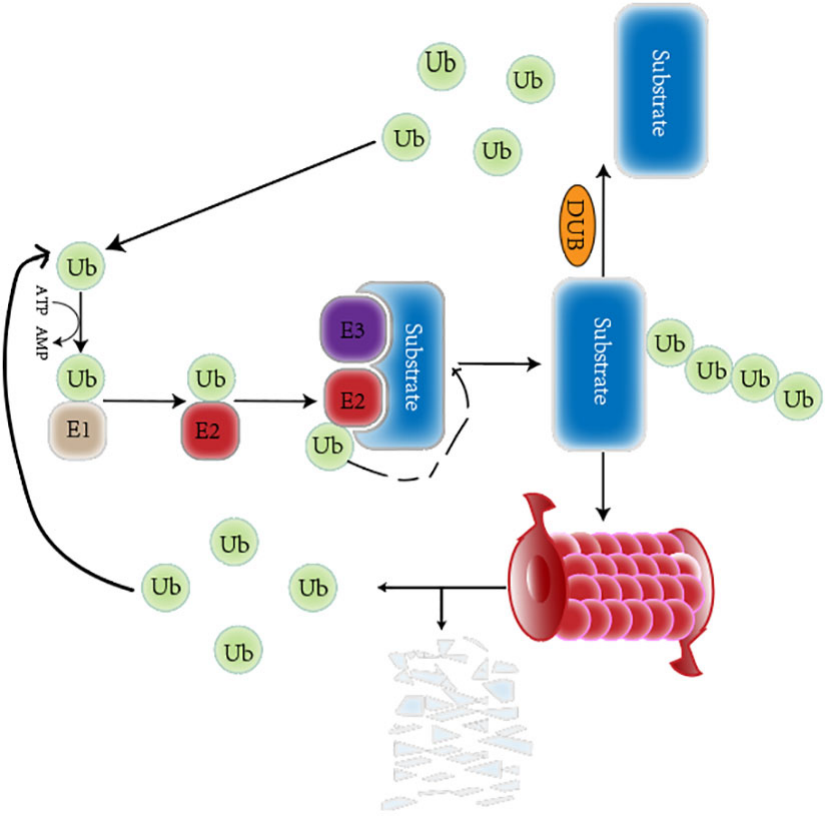
A brief overview of the ubiquitination and deubiquitination circulation. The Ub is respectively activated by the E1 Ub-activating enzyme, conjugated to the E2 Ub-conjugating enzyme, and attached to a specific substrate selected by E3 Ub ligase. The ubiquitinated proteins can be degraded by the proteasome. The deubiquitinase can remove Ub from substrate. Those released Ub participates in ubiquitination again.

The ubiquitin-proteasome system (UPS) is a critical PTM of proteins that regulates protein degradation and maintains protein homeostasis (16). The UPS consists of many key components, including ubiquitin(Ub), a highly conserved 76-amino-acid protein that conjugates other cellular proteins and modifies them (17). In addition, the UPS consists of deubiquitinating enzymes (DUBs) and a three-enzyme cascade involving ubiquitin-activating enzymes (E1s), ubiquitin-conjugating enzymes (E2s) and ubiquitin ligases (E3s) (18). Deubiquitination can remove mono Ub or the whole polyubiquitin chains on proteins to resist the protein’s degradation or the alteration of the subcellular position (19). Also included in the UPS is the 26S proteasome, which mainly consists of two parts: a 20S core particle (CP) complex, where protein degradation mainly occurs, and a 19S regulatory particle (RP), responsible for the substrate choosing the right degradation site (20). Usually, E1 activates Ub in an ATP-dependent manner. The activated Ub is then moved to E2 through a transthiolation reaction. The E3 and substrate protein complex acquire Ub from E2, and Ub targets the protein. Alternatively, Ub is moved to E3 through a transthiolation reaction, E3 recognizes the protein specifically, and the C-terminal of Ub is attached to the Lys residue of the target protein (21). In humans, two E1s, 38 E2s, and approximately 600–1000 E3s have been found so far (22). Studies have found that ubiquitination and deubiquitination reactions are responsible for developing various tumors and play key roles in cancer treatment (23). The ubiquitinization and deubiquitination process is shown in the **Figure 1**.

## 2 ncRNAs and NSCLC

NcRNAs participate in all aspects of NSCLC occurrence and development and play an essential role in NSCLC diagnosis and treatment. Parts of the crucial ncRNAs are enumerated in this review to explain their critical role in NSCLC.

### 2.1 miRNA and NSCLC

Dysregulation of miRNAs causes abnormal expression of target genes and influences every aspect of NSCLC. Numerous studies have verified that miRNAs influence NSCLC through cell growth, cell cycle, apoptosis, metastasis, immune escape, and drug resistance (24).

In 2004, let-7 was reported to have low expression in NSCLC causing cell growth inhibition, and was associated with shortened postoperative survival (25). The let-7 family inhibits tumor growth and metastasis in lung adenocarcinoma through the MAPK/ERK and Wnt/β-catenin pathways (26, 27). let-7 has a strong relationship with drug resistance by targeting different proteins or regulating other factors. Cancer stem cells (CSCs) have stem cell-like characteristics that enable the growth of tumor cells, which is closely related to tumor treatment resistance (28). Lin28 is a highly conserved RNA-binding protein that induces pluripotent stem cell differentiation. The double negative feedback loop established by let-7 and Lin28 significantly influences NSCLC chemotherapy and radiation therapy resistance (29, 30). A recent study found that metformin could increase mature let-7b expression by mediating m6A formation on pri-let-7b and enforce osimertinib sensitivity by decreasing the expansion of stem cell groups (31).

MiR-21 is one of cancer research’s first identified “miRNA enhancers” (32). All cells in the human body can produce extracellular vesicles (EVs), which are membranous vesicles released into the extracellular matrix. Exosomes are EVs with diameters ranging between 30 and 150 nm. The molecular content of exosomes, including nucleic acids, proteins, and lipids, has an important influence on various cells. Therefore, exosomal ncRNAs and their pathophysiological roles significantly impact NSCLC (33). MiR-21 was significantly higher in sputum and plasma samples from patients with NSCLC than healthy donors. Thus, in addition to promoting growth and invasion phenotypes, miR-21 is a non-invasive biomarker for NSCLC diagnosis (32, 34). Recent studies have focused on the combined effects of let-7 and miR-21. Bai et al. found that the simultaneous downregulation of miR-21 and upregulation of let-7 could inhibit NSCLC development. This finding indicates a feedback regulation loop between miR-21 and let-7, with K-Ras as the target gene (35). This study also looks at the combined effects of miRNAs, which could be significant in NSCLC research.

The characteristics of early metastasis make NSCLC survival rates extremely low. Epithelial-mesenchymal transition (EMT) is a typical change in the invasiveness and migratory abilities of tumor cells. Accompanied by converting epithelial cells to mesenchymal cells, epithelial cells lose cell-cell adhesion, have increased motility, and have altered the expression of some key genes (36). The miR-200 family includes five conserved miRNAs: miR-200a, miR-200b, miR-200c, miR-141, and miR-429. MiR-200 is a well-known tumor suppressor that inhibits EMT in various cancers. Previous studies have found that the typical EMT regulator, miR-200/ZEB1 axis, regulates ECM-dependent β1-integrin/FAK signaling in NSCLC through CRKL. In addition, previous studies further explored the intracellular signaling pathways in NSCLC responsible for tumor cell invasion and metastasis through the EMT activation (37). At the same time, a novel double-negative feedback loop between FOXF2 and miR-200 was discovered, revealing a parallel axis to miR-200/ZEB1 that controls cancer cell invasion and migration through the EMT (38). In addition to influencing invasion and metastasis, the EMT can also mediate drug resistance. A study found that Cathepsin L (CTSL) and miRNA-200c suppress each other and mediate paclitaxel resistance through EMT changes (39). Moreover, new research found that miRNA-200c could downregulate Lin28B and improve EMT-related EGFR tyrosine kinase inhibitor (TKI) resistance in NSCLC (40). MiR-200s and let-7s have multiple members that regulate overlapping target sets.

Three typical miRNAs in NSCLC and three research suggestions regarding the correlation between miRNAs and NSCLC are provided. First, early NSCLC diagnosis is critical for mortality reduction because more miRNAs function as exosomes. Second, miRNAs are prone to develop drug resistance during NSCLC treatment, as they can modulate drug resistance through many methods. Therefore, research should improve the efficiency of NSCLC treatment. Last but not least, miRNAs can be the enhancer or suppressor of NSCLC. In addition, several miRNAs have the same target genes. Thus, the overlapping effects of these miRNAs in NSCLC, which play a leading role, are expected to provide new insights into future research on NSCLC.

### 2.2 lncRNA and NSCLC

To date, numerous lncRNAs have been identified in NSCLC. Similarly, the effects of lncRNAs on the various stages of NSCLC development have received widespread attention. This paper clarifies the reciprocal of lncRNAs and NSCLC and future research directions by enumerating classic lncRNAs that have been identified.

The metastasis-associated lung adenocarcinoma transcript 1 (MALAT1), also known as nuclear-enriched abundant transcript 2 (NEAT2), is the first lncRNA to be studied in NSCLC. It is upregulated in tumor tissues of NSCLC and is considered a specific marker for NSCLC (41). LncRNAs can regulate the biological functions of NSCLC through many mechanisms; the most common regulating method is by acting as endogenous miRNA sponges. Several miRNAs are sponged by MALAT1 and regulate functions, including cell proliferation, differentiation, and development, through competing with mRNAs. For example, miR-1297 binding to MALAT1 was predicted using software and confirmed by luciferase reporter assays. P300 is the downstream target molecule of MALAT1 and miR-1297. The P300/β-catenin complex is essential for activating the Wnt pathway during transcriptional activity (42). In addition, the Wnt signaling pathway is closely related to drug resistance. Therefore, through inhibiting MALAT1/miR-1297/p300/β-catenin/Wnt signaling, the A549/DDP cells were re-sensitized. Asiatic acid can achieve this regulation (43). Similarly, the MALAT1/miR-27a-5p/PBOV1 axis was recently found to enhance gemcitabine resistance in NSCLC cells (44). In addition to being a ceRNA, MALAT1 is also involved in regulating NSCLC through other mechanisms. IGF2BP2 promotes MALAT1 stability *via* m6A modification and promotes the proliferation of NSCLC cells through the MALAT1/ATG12 axis (45). Furthermore, MALAT1 is an important circulating diagnostic biomarker for diagnosing NSCLC (46). The single nucleotide polymorphism (SNP) in MALAT1, rs3200401, has been associated with NSCLC susceptibility, which is essential for identifying early screening populations for NSCLC (47).

LncRNA H19 (referred to as H19) is another classic lncRNA that can influence NSCLC. LncRNA H19 is encoded by H19 protein and is highly expressed in NSCLC tissues and cells. FOXF2 has been certified as one of the promoters to accelerate H19 transcription. Upregulated H19 can promote the proliferation and migration of NSCLC through the EZH2/PTEN axis (48). H19 decreased the overall survival of NSCLC patients, the combination of H19 and miR-21 played important roles in diagnostic and treatments value in NSCLC (49). In addition, H19 can also act as a sponge for several microRNAs (miRNAs). For example, downregulating H19 can significantly inhibit EMT progression through the upregulation of miR-203 in NSCLC (50). As for drug resistance, related research found that H19 could be secreted into exosomes assisted by heterogeneous nuclear ribonucleoprotein A2/B1 (HNRNPA2B1) and induce gefitinib resistance in NSCLC cells (51). The latest study confirmed that exosomal H19 promotes erlotinib resistance *via* the miR-615-3p/ATG7 axis (52). H19 can also regulate the radiosensitivity of NSCLC cells. Radiotherapy (RT) is a crucial treatment modality. Theoretically, higher doses of radiation have a better effect on radiotherapy; however, related side effects may gradually appear. Therefore, it is essential to enhance radiosensitivity. Zhao et al. found that the H19 sponge miR-130a-3p increased the expression of WNK lysine deficient protein kinase 3 (WNK3) and elevated radiosensitivity to X-rays in NSCLC cells (53). Moreover, SNPs in H19 were associated with NSCLC susceptibility (54).

LncRNA-MEG3 is a representative inhibitory lncRNA in NSCLC. Tumor-suppressive lncRNAs inhibit carcinogenesis and progression by enhancing apoptosis *via* several methods. MEG3 induces apoptosis and inhibits proliferation by activating p53 (55). The upregulation of MEG3 inhibits stem cell-like characteristics and prevents metastasis in NSCLC through miR-650/SLC34A2 (56). As for therapeutic aspects, MEG3 might enhance cisplatin sensitivity *via* different signaling pathways (57, 58). Autophagy refers to a lysosome-mediated catabolic process and is a double-edged sword for NSCLC that can regulate the development of NSCLC through different proteins and signaling pathways. On the one hand, autophagy restrains tumorigenesis by removing harmful cytotoxic agents to reduce stress injury, prevent genome damage, and maintain cellular integrity (59). Wang et al. found that MEG3 influences autophagy by regulating the miR-543/IDO axis (60). On the other hand, the MEG3 rs4081134 polymorphism has been associated with NSCLC susceptibility in the Chinese population (61).

Considering the current research on lncRNAs and NSCLC, it can be concluded that lncRNAs regulate the progression of NSCLC through multiple mechanisms. Therefore, lncRNAs play a key role in the diagnosis and treatment outcomes of NSCLC. In addition, SNPs in a few classic lncRNAs are closely related to NSCLC susceptibility, which is of great significance for early lung cancer screening.

### 2.3 circRNA and NSCLC

Extensive research was only conducted on circRNAs after lncRNAs and miRNAs. However, due to their more stable characteristics, research on NSCLC and circRNAs is very prosperous, and the related mechanistic studies are more detailed and in-depth.

As mentioned above, exosomes can be used as diagnostic markers to regulate drug resistance and can be key factors in promoting the progression of NSCLC. More than 1000 exosomal circRNAs have been discovered in human serum, much more than linear RNAs. These exosomal circRNAs have a significant effect on NSCLC. For example, three circRNAs, circ_0047921, circ_0056285, and circ_0007761, were found as exosomal circRNAs in NSCLC patients’ serum exosomes, and could perfectly distinguish early-stage NSCLC and other lung diseases, such as pulmonary tuberculosis, in healthy people (62). Therefore, circRNAs have become excellent non-invasive diagnostic biomarkers for NSCLC. Moreover, several exosomal circRNAs have been found to regulate resistance to chemotherapy and radiotherapy in NSCLC. Exosomal circ_0001658 has been found to sponge miR-409-3p and increase TWIST1 expression to promote gefitinib resistance in NSCLC (63). The exosomal circUSP7 modulates the miR-934/SHP2 axis to induce anti-PD1 resistance and promote immune escape in NSCLC (64). Controlling tumor cell escape is crucial for improving radiotherapy sensitivity.

The tumor microenvironment (TME) includes a variety of cells and structures surrounding tumor cells. TME cells undergo metabolic modifications and regulate drug resistance (65). Recent studies have revealed the relationship between circRNAs and altered metabolism, which could assist in addressing the progression and drug insensitivity of NSCLC. The expression of circ_0008797 is low in NSCLC tissues and cell lines, which could attenuate proliferation, metastasis, and aerobic glycolysis by regulating miR-301a-3p/SOCS2 (66). The downregulation of circ_0011298 enhances the Taxol sensitivity of Taxol-resistant NSCLC cells by decreasing cell growth, metastasis, and glycolysis and promoting apoptosis and cell cycle arrest *via* the miR-486-3p/CRABP2 axis (67). In addition, circSLC25A16 and circPIP5K1A can induce hypoxia-inducible factor (HIF)-1a-dependent glycolysis by sponging miRNAs (68, 69).

The induction of programmed cell death (PCD) is the primary mechanism leading to tumor cell death. Pyroptosis, autophagy, and ferroptosis are novel and essential methods for inducing PCD. CircRNAs have an intimate relationship with PCD (70). A current study found that circDTL functions as an oncogene and the knockdown of circDTL could improve the efficacy of chemotherapy drugs, ferroptosis, and apoptosis of NSCLC cells through the miR-1287-5p/GPX4 axis (71). CircRNAs can regulate autophagy by directly targeting key proteins or signaling pathways related to autophagy. Hsa_circ_0085131 functions as a ceRNA and enhances cisplatin resistance of NSCLC cells by upregulating the autophagy-associated factor, ATG7, leading to autophagy of tumor cells (72). Similarly, circ_FOXM1 can lead to autophagy by sequestering miR-149-5p and upregulating ATG5 (73). In addition, some bioinformatic predictions have identified six circRNAs that regulate autophagy proteins by sponging miRNAs and enhancing the sensitivity of NSCLC to radiotherapy (74). Future experiments are expected to verify these predictions.

This paper did not enumerate the classic circRNAs to clarify their relationship with NSCLC. Although the research on circRNA is limited, many circRNAs have been found to influence NSCLC by regulating factors, including metabolism, TME, and autophagy. These are novel and popular NSCLC mechanism studies. Therefore, circRNAs are bound to be excellent research objects for diagnosing and treating NSCLC or exploring new mechanism targets and networks.

## 3 The UPS and NSCLC

During ubiquitination and deubiquitination in NSCLC, the E3 ligases and the DUBs play crucial roles in regulating protein stability that influences the progression of NSCLC. Therefore, E3 ligases and DUBs illustrated the relationship between ubiquitination, deubiquitination, and NSCLC.

### 3.1 E3 ligases and NSCLC

The superfamily of tripartite motif proteins (TRIM) are proteins containing an N-terminal RING finger, a coiled-coil (CC) domain, and one or two B-boxes (75). The RING domain is responsible for conjugations with ubiquitin or ubiquitin-like molecule, including interferon-stimulated gene15 (ISG15) and small ubiquitin-like modifier (SUMO). The C-terminal domains can categorize TRIM proteins and recognize and regulate substrate proteins (76). Many TRIM proteins have been found to regulate the development and treatment of NSCLC by targeting different proteins and signaling pathways (77). TRIM59 expression in NSCLC patients was two to three fold higher than in normal patients; these patients had worse survival outcomes suggesting that TRIM59 might be a novel biomarker for NSCLC diagnosis (78).

Similarly, DNA hypermethylation of TRIM58 was upregulated in NSCLC tissues and presented promising results in Area Under Curve (AUC) when discriminating between NSCLC and control groups (79). The TRIM family also affects drug resistance in NSCLC. TRIM46 promotes cell proliferation, glycolysis, and DDP in NSCLC by upregulating HK2 *via* AKT phosphorylation and PHLPP2 ubiquitination by interaction with TRIM46 is the key method for inducing p-AKT (80). TRIM23 was highly expressed in DDP-resistant lung adenocarcinoma (LUAD) cells and tissues. A mechanistic study suggested that TRIM23 could ubiquitinate proteins to activate the NF‐κB pathway and further regulate glucose metabolism in DDP cells (81). In addition to the TRIM family, many other classic E3 ligases participate in regulating NSCLC. Ubiquitin-conjugating enzyme E2O (UBE2O), an E2/E3 hybrid ubiquitin-protein ligase, targets MAX-interacting protein 1 (Mxi1) for ubiquitination and degradation at the K46 residue, which can suppress the occurrence of NSCLC and enhance radiosensitivity (82). Another E3 ligase, MIB1, stimulates the degradation of the antioxidant transcription factor, nuclear factor erythroid 2-related factor 2 (Nrf2), in a ubiquitin manner and induces them to be more sensitive to ferroptosis (83).

### 3.2 DUBs and NSCLC

Ubiquitin-specific proteases (USPs) are the most versatile class of known deubiquitinases with the most diverse structures that remove Ub from proteins. Among currently available research, USPs regulate multiple known NSCLC-related pathways (84). Knocking out the USP1 could lead to hypersensitivity DNA damage. Recent studies have found that inhibiting the activity of the USP1/UAF1 complex can improve cisplatin sensitivity in NSCLC (85). USP18 removes the conjugate of interferon-induced Ubl ISG15 and inhibits 14-3-3ζ acetylation by ISG15 to accelerate NSCLC metastasis (86). Additionally, USP35 targeted ferroportin and induced ferroptosis in NSCLC (87). USP5 increased PD-L1 levels by cleaving and stabilizing the polyubiquitin chain and maintaining stability. Thus, USP5 knockdown might prevent immune response and drug resistance by reducing PD-L1 protein (88). OTU deubiquitinase 3 (OTUD3) binds to the ovarian tumor-related protease family, PTEN protector. OTUD3 is a potential tumor suppressor in NSCLC, a deubiquitylase of GRP78, and promotes tumorigenesis of NSCLC (89). Ubiquitin C-terminal hydrolase-L3 (UCHL3) belongs to the ubiquitin COOH-terminal hydrolase family, and its high expression is associated with poor survival in LUAD. In addition, UCHL3 promotes the proliferation and stem cell traits of NSCLC cells by deubiquitinating Aryl hydrocarbon receptor (AhR) (90).

The number of E3 ligases and DUBs is enormous. They target key proteins in NSCLC by inducing ubiquitination or deubiquitination of these proteins or further regulating post-modifications of other proteins to regulate the progression of NSCLC. Therefore, the balance between ubiquitination and deubiquitination plays a crucial role in proliferation, metastasis, apoptosis, the cell cycle, immune escape, and drug resistance in NSCLC.

## 4 Interaction between UPS and regulatory ncRNAs in NSCLC

The ncRNA and the UPS systems have huge members and diverse control methods, which is why they became key regulators of NSCLC. Many studies focused on the interactions between ncRNAs and the UPS in NSCLC. However, compared to the vast number of these two systems, interaction research was fairly small. Recent studies have shown that ncRNAs could act on critical Ub-enzymes, other essential proteins, and pathways to regulate the development of NSCLC. Furthermore, certain ubiquitination and deubiquitination processes can conversely regulate ncRNAs. Such crosstalk between ncRNAs and ubiquitination or deubiquitination is significant and may even lead to breakthroughs in further NSCLC studies. Next, the necessity of research on crosstalk is illustrated by providing classic examples.

Several studies have been conducted on the let-7 family and the UPS. Histones, H2A and H2B, have monoubiquitination sites at their lysines, and K120 is the only site of H2B monoubiquitination that promotes the occurrence of H2Bub1. H2Bub1 functions as a tumor suppressor in lung cancer and can be catalyzed by the E3 ubiquitin-ligase complex, RNF20/RNF40, and erased by various DUBs (91). Ambra et al. performed a bioinformatic screen to identify the effects of miRNAs on H2Bub1 homeostasis. The results showed that let-7b targeted USP42, USP44, and ATXN7L3. USP42 and USP44 were reported to act on H2Bub1, while ATXN7L3 could remove H2Bub1 through the activity of USP22. Further experimental data showed that let-7b and let-7c significantly reduced the steady-state mRNAs and ATXN7L3 and USP42 protein levels but did not influence RNF20 protein levels. The results showed that the let-7 family members, especially let-7b and let-7c, could positively regulate H2Bub1. Further, RNA pull-down assays strongly supported that ATXN7L3, USP44, and USP42 were enriched by pulling down let-7b and were the direct let-7b targets. Altogether, these findings suggest that the let-7 family could prevent the ubiquitination of H2B by directly regulating DUBs to prevent the migration of NSCLC (92). Epidermal growth factor receptor (EGFR)-TKIs can induce drug resistance by elevating autophagic flux (93). One study found that miR-4487 expression increased in NSCLC cells treated with gefitinib. Subsequently, miR-4487 targeted USP37 directly and downregulated USP37 expression. These results led to whole-cell ubiquitination and increased autophagic flux, which also allowed the observation of miRNAs’ effects on NSCLC resistance by regulating deubiquitination and autophagy (94). Previous studies have suggested that KRAS mutations might promote tumor cell growth by inducing DNA damage and genotoxic stress. As a highly conserved recombinase, RAD51 may repair DNA damage caused by KRAS-MT and improve resistance to radiation (95). MiR-376a-5p acts as an upstream regulator of TRIM36; knockdown of miR-376a-5p upregulates TRIM36. Elevated levels of TRIM36 can inhibit DNA repair and promote radiosensitivity (96). Cullin 4B (CUL4B) belongs to the cullin family, which can assemble DNA damage-binding protein 1 (DDB1) and its substrate to form a new RING-based E3 ubiquitin ligase, cullin4B-Ring E3 ligase complex (CRL4B). MiR-194 can target CUL4B directly, inhibiting its translation and functions. On the other hand, CUL4B was important for H2AK119ub1 and EZH2 recruitment and the consequent H3K27me3 in miR-194. EZH2 repressed miR-194. This study described the role played by the negative feedback loop, including miR-194 and CUL4B. Negative feedback plays an essential role in NSCLC (97). More information on the effects of miRNAs interacting with the UPS in NSCLC is shown in **Table 1**.

**Table 1:** The interaction between miRNAs and UPS in NSCLC.

MIRNA	E3/*DUBS*	Target proteins	Reaction axis	Function	Reference
miR-138-5p	TRIM65	TNRC6A	TRIM65/TNRC6A/MiR-138-5P/ATG7	Promotes cisplatin resistance	(98)
miR-195/miR-497	SMURF2	TβRI	miR-195,miR-497/SMURF2/TβRI/TGF-β/SMAD	Promotes proliferation, invasion and xenograft tumor growth	(99)
miR-520b	SPOP	GLI2/3	miR-520b/SPOP/GLI2/3/Hh	Promotes proliferation and migration	(100)
miR-135b	*CYLD*	RIP1	IL-6/STAT3/miR-135b/*CYLD*/RIP1/NF-κB	Promotes migration, invasion, angiogenesis, xenograft tumor growth and anti-apoptosis	(101)
miR-101-3p	*USP47* MDM2	RPL11 P53	miR-101-3p/*USP47*/RPL11/MDM2/P53	Suppresses proliferation	(102)
miR-30-5p	*USP22*	HIF-1α	miR-30-5p/*USP22* /HIF-1α/PD−L1	Suppresses immune evasion	(103)
miR-320b	*USP37*	CDT1	miR-320b/*USP37*/CDT1	Suppresses proliferation, invasion and xenograft tumor	(104)
miR-365	*USP33*	ROBO1	miR-365/*USP33*/SLIT2-ROBO1	Promotes proliferation, migration, invasion and xenograft tumor growth	(105)
miR-489-3p	*USP48*	β-catenin	miR-489-3p/*USP48*/β-catenin/Wnt	Promotes proliferation, invasion, migration and xenografted tumor growth and metastasis	(106)

Some points regarding the interactions between miRNAs and the UPS can be concluded from the examples provided. Based on current research, miRNAs usually regulate the ubiquitination degradation or deubiquitination stabilization of key proteins through E3s or DUBs, which are the main methods that affect NSCLC development. Other studies have also revealed that E3s or DUBs could regulate miRNAs, partially forming a negative feedback loop. Such reaction networks play a critical role in the tumorigenesis, diagnosis, and treatment of NSCLC. Some miRNAs regulate the expression levels of target proteins and change their locations through E3s or DUBs, thus promoting or inhibiting the subsequent reactions. Studies on drug resistance and radiotherapy sensitivity of NSCLC influenced by miRNAs and the UPS are equally popular. Further miRNAs and E3s or DUBs interactions have been discovered, but further mechanistic research is required (107, 108). Thus, further research should lead to more spillovers in diagnosing and treating NSCLC.

The interactions between lncRNAs and E3s or DUBs are more complex than the interactions between miRNAs and the UPS. Tumor-associated macrophages (TAMs) are closely related to the TME and can be classified into M1 and M2 macrophages. M1 macrophages function as tumor suppressors, whereas M2 macrophages have the opposite effect. M2 polarization occurs more easily in the TME (109). In addition, exosomes and M2 macrophages can interact with each other to promote tumorigenesis (110). MiR-19b-3p could work as an exosomal miRNA and play an oncogenic role in NSCLC. On the one hand, exosomal miR‐19b‐3p promotes M2 polarization. Further, M2-polarized macrophages secrete exosomal LINC00273, which recruits neural precursor cell expressed developmentally downregulated 4 (NEDD4) and induces large tumor suppressor kinase 2 (LATS2) ubiquitination. NEDD4 is an E3 ligase that functions as an oncogene by promoting the ubiquitination of tumor-suppressive proteins (111). Therefore, miR‐19b‐3p regulates the Hippo/YAP pathway through LATS2 ubiquitination and promotes tumor progression. On the other hand, LINC00273 could also increase the RBMX level through Hippo/Yes associated transcriptional regulator (YAP). X-linked RNA-binding motif protein (RBMX) is an hnRNP that functions as an RBP to adjust the packaging of exosomal miRNAs. Therefore, RBMX helped package miR-19b-3p into LUAD cell-derived exosomes (112). This closed-loop plays an essential role in M2 polarization and LUAD development. This paper elucidates the crosstalk between TAMs and LUAD cells mediated by exosomes, ncRNAs, crucial pathways, and proteins, which provides new possibilities for LUAD diagnosis and treatment. The expression of UCHL3 was upregulated in NSCLC tissues and cells and is related to an unfavorable prognosis. Overexpression of LINC00665 and silencing of miR-582-5p enhanced the resistance of NSCLC cells to radiotherapy by upregulating UCHL3 and PD-L1 and stabilizing AhR to promote immune escape (113). Another study confirmed that lncRNA CCAT1 could activate and stabilize the PI3K/AKT/mTOR pathway. This stabilization is done by translocating fatty acid binding protein 5 (FABP5) into the nucleus to induce the PPAR‐RXR complex and pyruvate dehydrogenase kinase 1 (PDK1) translation. Further, it binds to USP49 and deubiquitinates FABP5, thereby binding to RAPTOR to induce AKT phosphorylation. CCAT1 participates in reprogramming FA metabolism and enhances the malignant phenotype of NSCLC (114). More information on the effects of lncRNA interactions with the UPS on NSCLC is shown in **Table 2**.

**Table 2:** The interaction between lncRNAs and UPS in NSCLC(when a protein acts as two roles, the two charts were merged).

LncRNAs	E3/*DUBS*	Ce/RBP/…	Target proteins	Reaction axis	Function	Reference
VAL	TRIM16	Vimentin		AKT/STAT3/VAL/Vimentin-TRIM16/EMT	Promotes invasion and migration	(115)
HOXC-AS3	MDM2	YBX1		HOXC-AS3/YBX1-MDM2/HOXC8	Promotes proliferation, migration, invasion, xenografted tumor growth and metastasis	(116)
OXCT1-AS1	NARF	LEF1		OXCT1-AS1/LEF1-NARF/EMT	Promotes migration, invasion and xenografted tumor metastasis	(117)
FAM83A-AS1	CRLs	HIF-1α		FAM83A-AS1/HIF-1α- VHL-CRLs	Promotes migration, invasion, stemness and glycolysis	(118)
LNBC3	FBXO11	BCL6		ASO/LNBC3/BCL6-FBXO11	Promotes migration and xenografted tumor growth	(119)
ALAL-1	*USP4*	SART3		ALAL-1/SART3-*USP4*/NF-κB	Promotes immune evasion and proliferation	(120)
GIAT4RA	*Uchl3*		LSH	GIAT4RA/LSH-*UCHL3*	Suppresses proliferation migration, invasion, xenografted tumor growth and metastasis	(121)
RMST	FBW7		SOX9	RMST/SOX9-FBW7	Suppresses proliferation, migration and xenografted tumor growth	(122)
LINC01426	*USP22*		SHH	LINC01426/*USP22*/SHH	Promotes proliferation, migration, and stemness but suppresses apotosis	(123)
lncKLF6	BMI1		H2A	lncKLF6/*USP22-*BMI1/KLF6	Promotes proliferation cell cycle, and xenografted tumor growth but suppress apotosis	(124)
SNHG16	*USP21*	miR-4500	YY1	*USP21*/YY1/SNHG16/miR-4500	Promotes proliferation, migration, and invasion	(125)
PKMYT1AR	SCF^β-TrCP^	miR-485-5p	β-catenin	ASO/PKMYT1AR/miR-485-5p/PKMYT1/SCF^β-TrCP^/β-catenin/WNT	Promotes proliferation, migration, cell cycle, cancer stem cell maintenance and chemo- or radio-therapy resistance, but suppress apotosis	(126)
SNHG12	*USP8*	HuR	PD-L1	SNHG12/HuR/*USP8*/PD-L1	Promotes proliferation and immune escape but suppress apotosis	(127)

Current research shows that the reactions between lncRNAs and the UPS are exceptionally complicated. In some studies, RBPs remained the target genes of the UPS. LncRNAs regulate ubiquitination or deubiquitination by competing with the binding sites of proteins or destroying the combination of proteins and E3s or DUBs. Some E3s or DUBs are RBPs, and lncRNAs can regulate enzymes directly, thus adjusting further reactions. Other lncRNAs regulate E3s or DUBs through their long miRNAs or RBPs. Some lncRNAs may influence multiple ubiquitination and deubiquitination processes. A few lncRNAs can alter the position of E3s and DUBs to regulate their effects. In conclusion, the diversity of ubiquitination or deubiquitination regulation by lncRNAs considerably impacts the occurrence and development of all stages of NSCLC. Whether it is TME, metabolism, SCS, immune escape, or other popular NSCLC research fields, crosstalk reactions can be seen everywhere. Therefore, further related studies should be conducted in the future.

The number of studies related to the regulation of circRNAs and the UPS is insufficient. Some studies have involved the regulation between E3s, DUBs, and circRNAs, but more profound mechanistic studies have not yet been conducted. Such research is relatively new and lacking, but it is crucial for the new direction of NSCLC diagnosis and treatment. CircIGF2BP3, a circRNA derived from the back-splicing of IGF2BP3, suppresses CD8T cell infiltration in NSCLC. Further, circIGF2BP3 is overexpressed and compromises anti-tumor immunity in NSCLC. METTL3 and METTL14 are composed of a stable methyltransferase complex (MTC) in a 1:1 ratio and exerts a methylation function (128). Promoting m6A levels in the circIGF2BP3 transcript in a METTL3-dependent manner could enhance the circularization of circIGF2BP3. In addition, circIGF2BP3 upregulates PKP3 expression in NSCLC cells by binding to miR-3173-5p or miR-328-3p. Plakophilin 3 (PKP3) belongs to the armadillo protein family, which can mediate PD-L1 expression and rescue it from proteasomal degradation. OTUB1 functions as a downstream effector of PKP3 and inhibits its degradation, thus further avoiding the killing effects of T cells and leading to immune escape by upregulating the PD-1 and PD-L1 complex (129). In this study, circIGF2BP3 and DUB became the PKP3 upstream and downstream, respectively, and the indirect effects of these two factors had an essential impact on the occurrence and development of NSCLC. Insulin-like growth factor-2 mRNA-binding proteins (IGF2BPs), IGF2BP1 and IGF2BP3, are evolutionarily conserved families of RNA-binding proteins that regulate important parts of cancer cells and promote the development of cancers (130). CircNDUFB2 has a length of 249 nucleotides and is generated from NDUFB2. Functionally, circNDUFB2 serves as a suppressor of NSCLC development. CircNDUFB2 enhances the interactions between TRIM25 and IGF2BPs and subsequently boosts the IGF2BPs ubiquitination by TRIM25. Interestingly, circNDUFB2 exerts a tumor-suppressive role by promoting IGF2BPs ubiquitination and eliciting immune responses in NSCLC cells. Retinoic acid-inducible gene I (RIG-I) is a member of the RIG-I-like receptor (RLR) family, which recognizes viral RNAs and induces innate immune responses against viral infections. When lacking an RNA ligand, RIG-I adopts an auto-repressed conformation that prevents the N-terminal caspase recruitment domains (CARDs) from signaling. CircNDUFB2 activates RIG-I by destabilizing the interaction between CARDs and its helicase domain, thereby inducing the activation of RIG-I-MAVS signaling cascades (131). In addition, circNDUFB2 promotes IGF2BPs ubiquitination through the circNDUFB2/TRIM25/IGF2BPs signaling pathway and causes cellular immune responses by activating RIG-I. CircNDUFB2 inhibited tumorigenesis by two mechanisms, which prompted a focus on the effects of circRNAs on tumors from multiple perspectives.

Most circRNAs regulated the E3s or DUBs by sponging miRNAs. Therefore, it can be said that the research on the crosstalk between the circRNAs and the UPS is in its infancy. However, with further development, crosstalk studies between circRNAs and the UPS are bound to bring more remarkable progress in diagnosing and treating NSCLC.

## 5 Conclusion

This article reviews the current research progress on the effects of interactions between ncRNAs and ubiquitination (and deubiquitination) in NSCLC. The intrinsic correlation between regulatory ncRNAs and UPS in NSCLC has been increasingly studied in recent years. It is worth noting that the interactions between ncRNAs and the UPS have been found to influence the progression of NSCLC, which makes them critical therapeutic targets. However, there is limited research that uncovers the precise mechanisms. There are many hurdles to overcome in studying the interplay between regulatory ncRNAs and the UPS in NSCLC. Nevertheless, with the continuous innovations of experimental methods and techniques, interactions between ncRNAs and ubiquitination and deubiquitination to innovate diagnosis methods and improve treatment efficiency in NSCLC are foreseeable.

## Author contributions

YS wrote the draft and revised it. PH designed and supervised the study. LL and XD collected the data and designed the figures. All authors contributed to the article and approved the submitted version.
